# Different onset patterns of monthly paliperidone palmitate in hospitalised patient diagnosed with schizophrenia

**DOI:** 10.1192/bjo.2021.459

**Published:** 2021-06-18

**Authors:** Javier Herrera-Sánchez, Julia Aznar, Leire Izaguirre, Santiago Ovejero

**Affiliations:** Hospital Universitario Fundación Jiménez Díaz

## Abstract

**Aims:**

Paliperidone palmitate long-acting injectable (PPLAI) initially requires two loading doses of 150 and 100 mg on days 1 and 8 (± 4 days) intramuscularly. In clinical practice, different PPLAI initiation patterns have been observed. The aim of this study is to describe different PPLAI onset patterns in hospitalised patients diagnosed with schizophrenia.

**Method:**

A naturalistic, transversal, retrospective, descriptive study was carried out. Patients were recruited in the adult inpatient unit of Hospital Universitario Jiménez Díaz (Madrid, Spain) from November 2012 to February 2021. During this period, a total of 357 patients were treated with PPLAI, 172 of them were diagnosed with schizophrenia and, among these, 24 received an atypical onset pattern during hospitalization. Different PPLAI onset patterns, PPLAI dose at discharge and number of days hospitalised were analysed. This study followed the Declaration of Helsinki principles and was approved by the Local Ethics Committee. All participants gave written informed consent.

**Result:**

The sample presents 24 patients (17 men, 7 women) that represents 6.72% of a global sample, with an average age of 40.21 years (men 35.59 years vs. women 51.43 years). In this study, different PPLAI onset patterns were described: those receiving 150-150 mg represent 25% of the sample (n = 6), as do those receiving 100-75 mg, also representing 25% of the sample (n = 6). The rest of the onset patterns were: 150-75 mg (20.83%, n = 5), 100-100 mg (12.5%, n = 3), 150-75 mg (4.16%, n = 1), 100-50 mg (4.16%, n = 1), 75-100 mg (4.16%, n = 1), and 75-75 mg (4.16%, n = 1). The average hospital stay is 17.88 days. The PPLAI maintenance dose at discharge was 104.17 mg/month. The group of patients who received two doses of 150 mg (150-150 mg) had an average length of stay of 27.67 days compared to the rest of the patients who had an average length of stay of 15.12 days, this difference being statistically significant (p = 0.010). The 150-150 mg group was discharged with a mean maintenance dose of 141.67 mg versus the other patients who needed a mean maintenance dose of 91.18 mg, which was also statistically significant (p = 0.001).

**Conclusion:**

The most used pattern of atypical onset of PPLAI in this sample is 150-150 mg and 100-75 mg. Patients treated with 150-150 mg loading pattern are hospitalized for a longer period and needed higher maintenance dose at discharge. Further studies are needed.

